# Lamprey Immune Protein Mediates Apoptosis of Lung Cancer Cells Via the Endoplasmic Reticulum Stress Signaling Pathway

**DOI:** 10.3389/fonc.2021.663600

**Published:** 2021-07-07

**Authors:** Xiaoping Song, Xiangting Xu, Jiali Lu, Xiaoyuan Chi, Yue Pang, Qingwei Li

**Affiliations:** ^1^ College of Life Science, Liaoning Normal University, Dalian, China; ^2^ Lamprey Research Center, Liaoning Normal University, Dalian, China; ^3^ Respiratory Medicine, Affiliated Zhong shan Hospital of Dalian University, Dalian, China

**Keywords:** lamprey, LIP, lung cancer, cytotoxicity, endoplasmic reticulum stress, apoptosis

## Abstract

Lamprey immune protein (LIP), a novel protein derived from the *Lampetra japonica*, has been shown to exert efficient tumoricidal actions without concomitant damage to healthy cells. Our study aimed to ascertain the mechanisms by which LIP inhibits lung cancer cells, thus delineating potential innovative therapeutic strategies. LIP expression in lung cancer cells was evaluated by western blotting and immunohistochemistry. Functional assays, such as high-content imaging, 3D-structured illumination microscopy (3D-SIM) imaging, flow cytometry, and confocal laser scanning microscopy, were performed to examine the proliferation and lung cancer cell apoptosis. Tumor xenograft assays were performed using an *in vivo* imaging system. We observed that LIP induces the decomposition of certain lung cancer cell membranes by destroying organelles such as the microtubules, mitochondria, and endoplasmic reticulum (ER), in addition to causing leakage of cytoplasm, making the maintenance of homeostasis difficult. We also demonstrated that LIP activates the ER stress pathway, which mediates lung cancer cell apoptosis by producing reactive oxygen species (ROS). In addition, injection of LIP significantly retarded the tumor growth rate in nude mice. Taken together, these data revealed a role of LIP in the regulation of lung cancer cell apoptosis *via* control of the ER stress signaling pathway, thus revealing its possible application in lung cancer treatment.

## Introduction

Lung cancer is the most common cause of cancer-related mortality worldwide, accounting for nearly 20% of all cancer deaths (1, 2). Human lung adenocarcinoma, human small-cell lung cancer, and human lung squamous cell carcinoma are the three common subtypes of lung cancer. In recent years, immunotherapy has played a pivotal role in the treatment of lung cancer. Targeting tyrosine kinase inhibitors has proven to be effective for patients with epidermal growth factor receptor (EGFR) mutations or anaplastic lymphoma kinase (ALK), ret proto-oncogene (RET), and receptor tyrosine kinase (ROS1) fusion (3). Programmed death-1 (PD-1) inhibitors also showed considerable efficacy in a small group of patients, in whom the tumor cells expressed high levels of the programmed cell death-ligand 1 (PD-L1) protein (4). Despite considerable breakthroughs in targeted therapy over recent years, a large proportion of lung cancer patients are compelled to undergo traditional chemotherapy and radiotherapy, resulting in a five-year survival rate of <20% (5, 6). In addition, targeted therapy is hindered by cell resistance, which ultimately leads to decreased drug sensitivity and relapse. Therefore, the development of more efficacious therapeutic approaches is essential for lung cancer.

Apoptosis or programmed cell death is often induced by chemotherapeutic agents and plays a critical role in the clinical treatment of human cancers. It is regulated by several apoptosis-related genes and signaling pathways, including inositol-requiring transmembrane kinase/endonuclease-1 (IRE-1)/palmitate-induced splicing of X box–binding protein-1 (sXBP-1) and pancreatic ER kinase (PERK)/CCAAT/enhancer-binding protein homologous protein (CHOP) (7–9). Lamprey immune protein (LIP), first isolated from the lamprey, is a 313-amino acid-long protein with an N-terminal jacalin-like domain and a C-terminal aerolysin domain (10, 11). In our previous study, LIP was shown to exert cytocidal effects on a variety of tumor cells, with minimal toxicity in normal cells (11). In addition, we explored the primary mechanism of LIP that effectively distinguishes tumor cells from normal cells, i.e., its ability to recognize biantennary non-fucosylated N-glycan or sialyl Lewis X-containing glycan structures in tumor cells (11). These studies revealed the potential of LIP for application in tumor diagnosis and treatment; however, the molecular mechanism underlying the inhibition of tumor growth by LIP remains unclear. In the present study, we elucidated the role of LIP in the regulation of tumorigenesis and apoptosis *via* the ER stress signaling pathway.

## Materials and Methods

### Animal and Cell Culture

Animal experiments were performed in accordance with the regulations of the Animal Welfare and Research Ethics Committee of the Institute of Dalian Medical University’s Animal Care protocol (permit number: SCXK2008-0002).

Human lung adenocarcinoma cell lines A549 and A549-Luc-C8, human small-cell lung cancer cell line NCI-H446, human lung squamous cell carcinoma cell line NCI-H520, and human embryonic lung epithelial cell line L132 were obtained from the American Type Culture Collection (ATCC; Manassas, VA, USA). Cells were cultured in Roswell Park Memorial Institute (RPMI) 1640 medium (Gibco, Grand Island, NY, USA) in a humidified incubator under 5% CO_2_ at 37°C. All media were supplemented with 10% (v/v) fetal bovine serum (FBS; Gibco, Grand Island, CA, USA), 100 μg/ml streptomycin, and 100 U/ml penicillin (Beyotime Biotechnology, Shanghai, China).

### Detection of Cell Mortality

Approximately 1 × 10^4^ NCI-H446, NCI-H520, A549, and L132 cells were seeded per well of a CellCarrier 96-well plate (PerkinElmer, Waltham, MA, USA) and allowed to attain confluence in a humidified incubator under 5% CO_2_ at 37°C for 24 h before treatment. Cells were washed twice with phosphate-buffered saline (PBS) and then transferred into RPMI 1640 medium containing 0.1% FBS. The cells were treated with 0, 1, 2, or 4 μM LIP for 24 h in a humidified incubator under 5% CO_2_ at 37°C. The cells were then washed thrice with PBS and subsequently stained with Hoechst 33342 (1 μg/ml; Sigma-Aldrich, St. Louis, MO, USA) and propidium iodide (PI; 5 μg/ml; Sigma-Aldrich, St. Louis, MO, USA) in PBS for 15 min at room temperature (about 20°C) in the dark. After washing with PBS, the cells were imaged with the Operetta™ High-Content Imaging System (PerkinElmer, Waltham, MA, USA) at 20× magnification and analyzed using the PerkinElmer Harmony software.

### Mitochondrial, ER, and Microtubulin Staining

A total of 5 × 10^4^ L132, NCI-H446, NCI-H520, and A549 cells were plated in confocal dishes (cover glass-bottom dishes) and treated with 2 μM LIP at 37°C for 24 h. The cells were then washed twice with PBS and stained with 1 μg/ml Hoechst 33342 at 37°C for 20 min to visualize the cell nuclei. Following this, the cells were washed twice with PBS and stained with 100 nM MitoTracker™ mitochondrion-selective probes or 1 μM ER-Tracker™ Dyes (Invitrogen, Carlsbad, CA, USA) in the dark at 37°C for 30 min. The samples treated with/without LIP were washed with PBS and then analyzed using a Zeiss LSM 780 inverted microscope (Carl Zeiss, Jena, Germany).

A total of 5 × 10^5^ NCI-H446, NCI-H520, and A549 cells were seeded into the respective 12-well plates crawling tablets and treated with 2 μM LIP at 37°C for 24 h. The cells were then washed twice with PBS, following which 4% paraformaldehyde was slowly added to the plates at room temperature for 20 min. Next, the cells were removed and permeabilized with 0.1% Triton X-100 in PBS at room temperature for 10 min before using 5% donkey serum to seal them at room temperature for 3 h. α-Tubulin monoclonal antibody (1:1,000, Thermo Fisher Scientific, San Jose, CA, USA) was added to the cells at 37°C for 1.5 h, after which the cells were incubated with donkey anti-mouse IgG H&L (Alexa Fluor^®^ 647) (Abcam, Cambridge, MA, USA) at room temperature in the dark for 45 min. Cell nuclei were counter-stained with 1 μg/ml DAPI (Thermo Fisher Scientific, San Jose, CA, USA) for 15 min. After washing the cells with PBS and ddH_2_O, their images were acquired using a DeltaVision OMX V4 imaging system (GE Healthcare, Madison, WI, USA).

### LDH Cell Mortality Assay

A total of 1 × 10^4^ NCI-H446, NCI-H520, A549, and L132 cells/well were seeded into the respective CellCarrier 96-well plates and allowed to attain confluence in a humidified incubator under 5% CO_2_ at 37°C for 24 h. The cells were subsequently washed twice with PBS and treated with 0, 1, 2, or 4 μM LIP in a humidified incubator under 5% CO_2_ at 37°C for 24 h. Lactate dehydrogenase (LDH) release was detected using the LDH Assay Kit (Abcam, Cambridge, MA, USA), while the LDH release rate in the cell culture supernatant was detected according to the manufacturer’s instructions. The cell culture plates were centrifuged at 400×*g* for 5 min, after which the supernatants (10 μl/well) were extracted into another 96-well plate. Subsequently, 45 μl LDH reaction mix was added to each well and incubated at room temperature for 30 min. The absorbance values were measured at 490 nm on a microplate reader (Bio-Rad, Hercules, CA, USA). The prismatic diagram was plotted using Prism 7 software (GraphPad Software, San Diego, CA, USA).

### Wound-Healing Assay

A total of 5 × 10^4^ NCI-H446, NCI-H520, A549, and L132 cells were grown overnight in 48-well plates. Cells were wounded by scratching with pipette tips and washed twice with PBS, after 0.5, 1, 2, or 4 μM LIP was added to the 48-well culture dishes. An inverted microscope (TE2000; Nikon, Tokyo, Japan) was used to capture images of the cells at 40× magnification after incubation for 24 h. Cell migration from the edge of the injured monolayer was quantified by measuring the distance from the wound edges. Cells that received only medium served as a negative control. Each experiment was performed in triplicate.

### Immunofluorescence Staining

A total of 5 × 10^4^ L132, NCI-H446, NCI-H520, and A549 cells were plated onto confocal dishes (cover glass-bottom dishes) and treated with 2 μM LIP at 37°C for 24 h. The cells were then washed twice with PBS and stained with Annexin V-FITC for 15 min at room temperature, according to the manufacturer’s instructions. This was followed by visualization of the cells using a Zeiss LSM 780 inverted microscope.

### Annexin V-FITC/Propidium Iodide Apoptosis Assay

The Annexin V-FITC Apoptosis Detection Kit (BD Pharmingen, San Diego, CA, USA) was used to detect apoptosis in the cells. Approximately 1 × 10^6^ L132, NCI-H446, NCI-H520, and A549 cells were collected and washed twice with ice-cold PBS after treatment with 2 or 4 μM of LIP for 24 h. The cell pellets were then resuspended in binding buffer and stained with Annexin V and PI, according to the manufacturer’s instructions. Apoptosis was analyzed using flow cytometry with a FACSCalibur flow cytometer (BD Biosciences, San Jose, CA, USA).

### Western Blotting

A total of 1 × 10^6^ L132, NCI-H446, NCI-H520, and A549 cells were seeded into 6-well plates and treated with 0, 2, or 4 μM LIP for 24 or 48 h. After treatment, the cells were harvested at various time intervals and digested in RIPA buffer in the presence of protease inhibitor (Pierce Biotechnology, Rockford, IL, USA) and protein phosphatase inhibitor (New Cell & Molecular Biotech, Suzhou, China) cocktails. Total protein was extracted and quantified using the BCA Protein Assay Kit (Pierce Biotechnology). Next, a 30 μg aliquot of protein from each sample was separated using SDS-PAGE and transferred to a PVDF membrane (Tanon, Shanghai, China). After blocking with 5% non-fat milk in TBST for 1 h at room temperature, the bands were incubated overnight at 4°C with the following specific primary antibodies: Anti-PARP1 antibody (1:1,000, Abcam, Cambridge, MA, USA), Anti-GRP94 antibody (1:1,000, Abcam), Anti-GRP78 Bip antibody (Bip, 1:1,000, Abcam), Anti-Caspase-12 antibody (1:2,000, Abcam), Anti-Caspase-3 antibody (1:5,000, Abcam), anti-CHOP (1:1,000, Proteintech Group, Chicago, IL, USA), and Anti-p21 antibody (1:1,000, Abcam). Anti-β-actin (1:1,000, Sigma-Aldrich, St. Louis, MO, USA) was used as an internal control in the western blotting analysis. The secondary antibody consisted of horseradish peroxidase (HRP)–conjugated goat anti-rabbit IgG (1:5,000, Proteintech Group, Chicago, IL, USA). Enhanced chemiluminescence (Beyotime Biotechnology, Shanghai, China) was used for immunoblot protein detection.

Moreover, 1 × 10^6^ NCI-H446, NCI-H520, A549, and L132 cells were seeded into 6-well plates, grown overnight, and then treated with a dose of 2 mM ER stress inhibitor 4-phenylbutyric acid (4-PBA) (MedChemExpress, Monmouth Junction, NJ, USA) and 4 μM LIP for 48 h. The control group was not treated with 4-PBA. Cell lysis and sample preparation for western blotting were performed using the same method as described above.

### Measurements of Mitochondrial Membrane Potential

The MMP was evaluated using the JC-1 MMP Detection Kit (Beyotime Biotechnology, Shanghai, China). In brief, CCCP (1:1,000, Abcam, Cambridge, MA, USA) was added as a positive control for 30 min under a humidified atmosphere of 5% CO_2_. After L132, NCI-H446, NCI-H520, and A549 cells were seeded into 6-well plates and treated with 2 or 4 μM LIP for 24 h, the cells were incubated with 1× JC-1 staining solution for 20 min at 37°C and then rinsed twice with 1× staining buffer. Finally, the cells cultured in RPMI 1640 with 10% FBS were detected using a FACSCalibur flow cytometer.

### Intracellular Reactive Oxygen Species Assay

A total of 5 × 10^4^ L132, NCI-H446, NCI-H520, and A549 cells were plated in the respective confocal dishes (cover glass-bottom dishes) and treated with 2 μM LIP at 37°C for 24 h. The cells were then washed twice with PBS and incubated with 10 nM 2,7’-dichlorofluorescein diacetate (Sigma-Aldrich, St. Louis, MO, USA) in the dark at 37°C for 30 min, according to the manufacturer’s instructions. The cells were subsequently stained with 1 μg/ml Hoechst 33342 at 37°C in the dark for 15 min. Cell staining was examined using a Zeiss LSM 780 inverted microscope.

### Assay for Synergistic Cytotoxic Effect of Cisplatin (DDP) and LIP Using the Operetta™ High Content Imaging Analysis System

Approximately 1 × 10^4^ NCI-H446, NCI-H520, A549, and L132 cells were seeded per well of a CellCarrier 96-well plate and allowed to attain confluence in a humidified incubator under 5% CO_2_ at 37°C for 24 h before treatment. Cells were washed twice in PBS and then transferred to RPMI 1640 medium containing 0.1% FBS. The cells were treated with 0, 0.5, and 4 μM LIP in a humidified incubator under 5% CO_2_ at 37°C for 24 h, in combination with/without 30 μM cisplatin (DDP). The cells were then washed thrice with PBS and subsequently stained with 1 μg/ml Hoechst 33342 and 5 μg/ml PI in PBS for 15 min at room temperature in the dark. After washing with PBS, the cells were imaged using the Operetta High-Content Imaging System at 20× magnification and analyzed using the PerkinElmer Harmony software.

### Tumor Xenograft Assay

Six-week-old male BALB/c nude mice were used to establish xenografts. For each injection, 1 × 10^6^ A549-Luc-C8 cells were collected and resuspended in 100 μl of ice-cold 20% Matrigel (BD Biosciences, San Jose, CA, USA) in PBS. This 100 μl solution was injected subcutaneously into the thighs of these nude mice using a 22-gauge needle. The transplanted A549-Luc-C8 cells were allowed to grow for 2 weeks, following which the xenografts with A549-Luc-C8 cells were divided into two groups according to the tumor volume, each group with 13 male nude mice. The two groups of animals received intratumoral injections of PBS or LIP (20 μg/kg) into the tumor sites every 2 days. Mice were monitored using the IVIS Lumina Series III *In Vivo* Imaging System (PerkinElmer, Waltham, MA, USA) twice weekly. Tumor length and width were measured using calipers, following which the tumor volumes were calculated using the equation (L × W^2^)/2, where L is the length of the tumor and W is the width of the tumor. Human tumor xenografts in the nude mouse model were allowed to grow for a month after injection. At the end of the experiment, the animals (n = 13) were sacrificed, their tumors were separated from the surrounding muscles, weighed, and analyzed using Prism 7 software.

### Immunohistochemistry

The most typical areas of lung adenocarcinoma tissues fixed with 4% paraformaldehyde (Sigma-Aldrich, St. Louis, MO, USA) were selected to construct frozen sections (4 µm thickness). Hematoxylin–eosin (HE) staining and immunohistochemical analysis of the sections were performed as previously described. Briefly, gradient ethanol was used to deparaffinize the sections. Antigen unmasking was performed using citrate buffer (pH 6.0), and endogenous peroxidase blocking was performed using 3% hydrogen peroxide. The slides were then washed with PBS and incubated with 1 μM LIP at 4°C overnight. A PV-9000 two-step method was performed in accordance with the manufacturer’s instructions (ZSGB-BIO, Beijing, China) using LIP monoclonal antibody as the primary antibody at a working dilution of 1:500 at 37°C for 3 h. After washing with PBS, secondary antibody reactions were carried out using HRP-conjugated goat anti-mouse IgG for 15 min. The reaction was visualized by treatment with ZLI-9018 DAB Kit (ZSGB-BIO, Beijing, China). The optimal incubation time of the DAB reaction was observed, following which the slices were immediately washed, dehydrated using a gradient concentration of ethanol, and finally placed in xylene. The recognition binding sites of LIP to the tumor tissues and nuclei were labeled with DAB (brown) and Hoechst (blue), respectively. The slices were visualized using a ZEN Blue Lite microscope (Carl Zeiss, Oberkochen, Germany).

### Statistical Analysis

Basic statistical analysis was performed using a GraphPad Prism version 7 for Windows (GraphPad Software, CA, USA). The results were presented as the mean ± standard deviation (SD) of independent triplicate experiments with three repeats. Statistical differences between groups were assessed using a Student’s t-test (two-tailed) analysis. ^*^P < 0.05 was considered statistically significant, and ^**^P < 0.01, ^***^P < 0.001 were considered highly significant.

## Results

### LIP Inhibited Cell Viability and Cell Migration in Human Lung Cancer Cells

**Figure 1:**
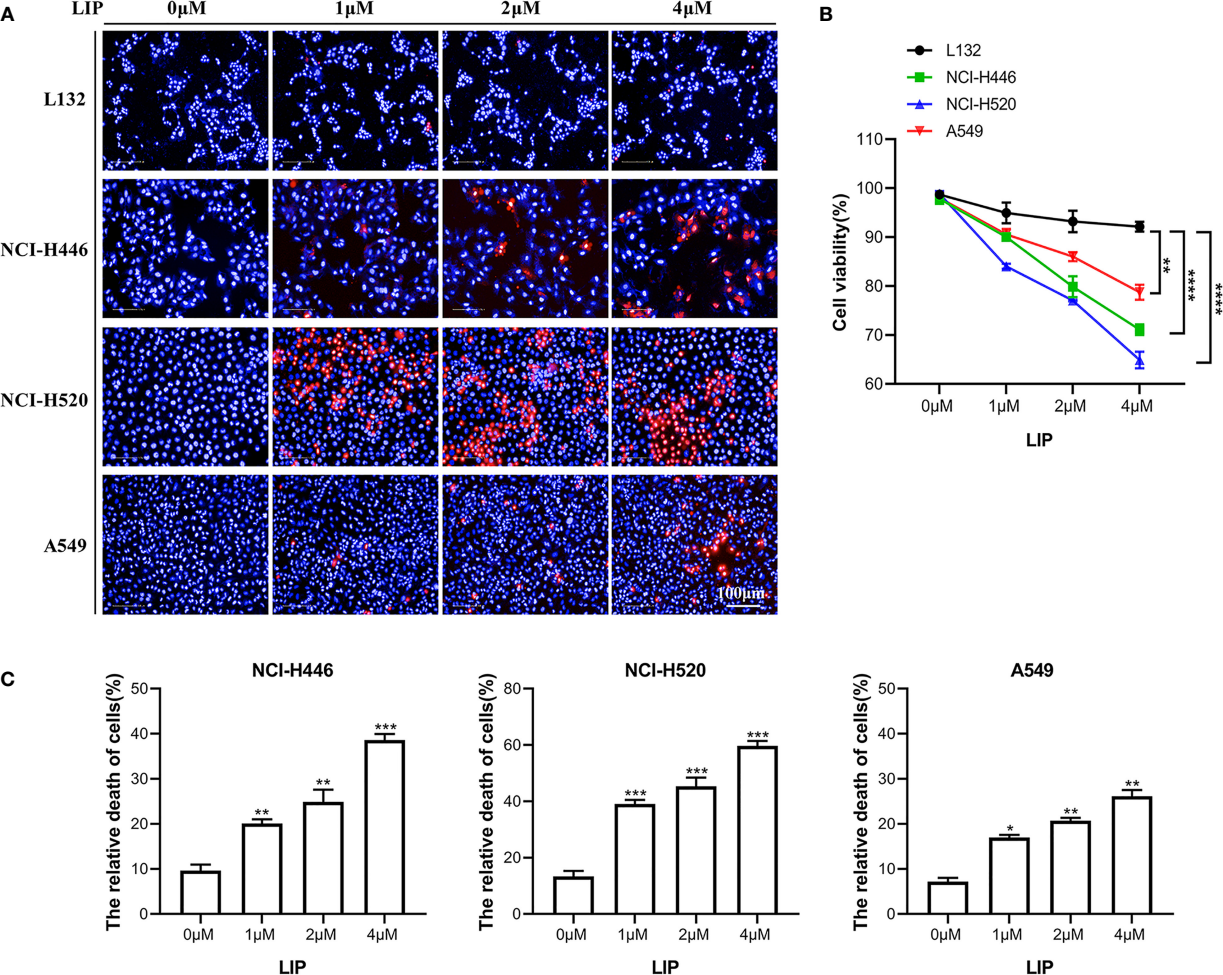
Sensitivity of NCI-H446, NCI-H520, A549, and L132 cell lines toward recombinant LIP. **(A)** Detection of sensitivity of the four cell lines toward LIP using the Operetta™ High-Content Imaging Analysis System. Scale bar: 100 µm. **(B)** Statistical analysis of mortality in the four cell lines. Student’s t-test was used for data analysis; ns, not significant; *P < 0.05, **P < 0.01, as compared to the control group (n = 3). **(C)** Cells were cultured with various concentrations of LIP for 24 h, following which the cell mortality was detected using an LDH assay. Student’s t-test was used for data analysis; *P < 0.05, **P < 0.01 and ***P < 0.001, as compared to the control group (n = 3).

Three lung cancer cell lines (small-cell lung cancer cell line NCI-H446, lung squamous cell line NCI-H520, and lung adenocarcinoma cell line A549) and a human embryonic pulmonary epithelial cell line (L132) were analyzed using the Operetta™ High Content Imaging Analysis System, to statistically estimate changes in mortality after LIP treatment. After 24 h of treatment, the cells were stained with PI and Hoechst 33342 and then observed using a high-content imaging system. PI staining was denoted by red, while Hoechst 33342 staining was denoted by blue. As seen in **Figure 1A**, there was a reduction in the viability of NCI-H446, NCI-H520, and A549 cells with increasing LIP concentration. As shown in **Figure 1B**, NCI-H446 and NCI-H520 cells were more sensitive, A549 cells were less sensitive, and L132 cells were not sensitive to LIP.

To further verify the sensitivity of NCI-H446, NCI-H520, and A549 cells to LIP, an LDH assay was used to assess cell viability. The results obtained after incubation of the three cell lines with different concentrations of LIP for 24 h are shown in **Figure 1C** (n = 3). With an increase in LIP concentration, there was a corresponding increase in the mortality rate of NCI-H446, NCI-H520, and A549 cells. Overall, after treatment with 4 μM LIP, A549 showed the lowest mortality rate of 26.7%, while NCI-H520 showed the highest mortality rate of 61.3%, consistent with the statistical results shown in **Figure 1B**.

**Figure 2:**
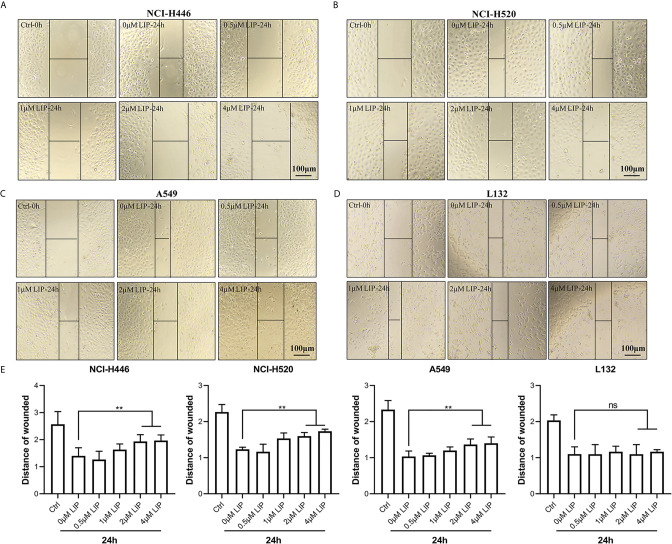
LIP inhibits cell migration in NCI-H446, NCI-H520, and A549 cell lines. **(A–C)** LIP remarkably inhibited NCI-H446, NCI-H520, and A549 cell migration in the wound-healing assay. Scale bar: 100 µm. **(D)** LIP did not inhibit L132 cell migration in the wound-healing assay. Scale bar: 100 µm. **(E)** Statistical analysis of migration in the four cell lines. Student’s t-test was used for data analysis; ns, not significant; **P < 0.01, as compared to the control group (n = 3).

To investigate whether LIP impacts cell migration, we conducted a wound-healing assay. It was found that low concentration of LIP did not affect the migration of lung cancer cell, but when the concentration of LIP reached 2 μM, it began to inhibit migration of NCI-H446 (**Figure 2A**), NCI-H520 (**Figure 2B**), and A549 (**Figure 2C**) cells. Conversely, it did not inhibit migration in L132 cells at the same dose (**Figure 2D**). Statistical analysis of the migration of the four cell lines was shown in **Figure 2E**.

### LIP Destroyed the Organelle Structure of Lung Cancer Cell Lines

To investigate the effects of recombinant LIP on the tissue structure of three lung cancer cell lines, namely NCI-H446, NCI-H520, and A549, we performed immunofluorescence experiments on the mitochondria, ER, and microtubule and observed the organelles using 3D-structured illumination microscopy (3D-SIM) and confocal microscopy.

**Figure 3:**
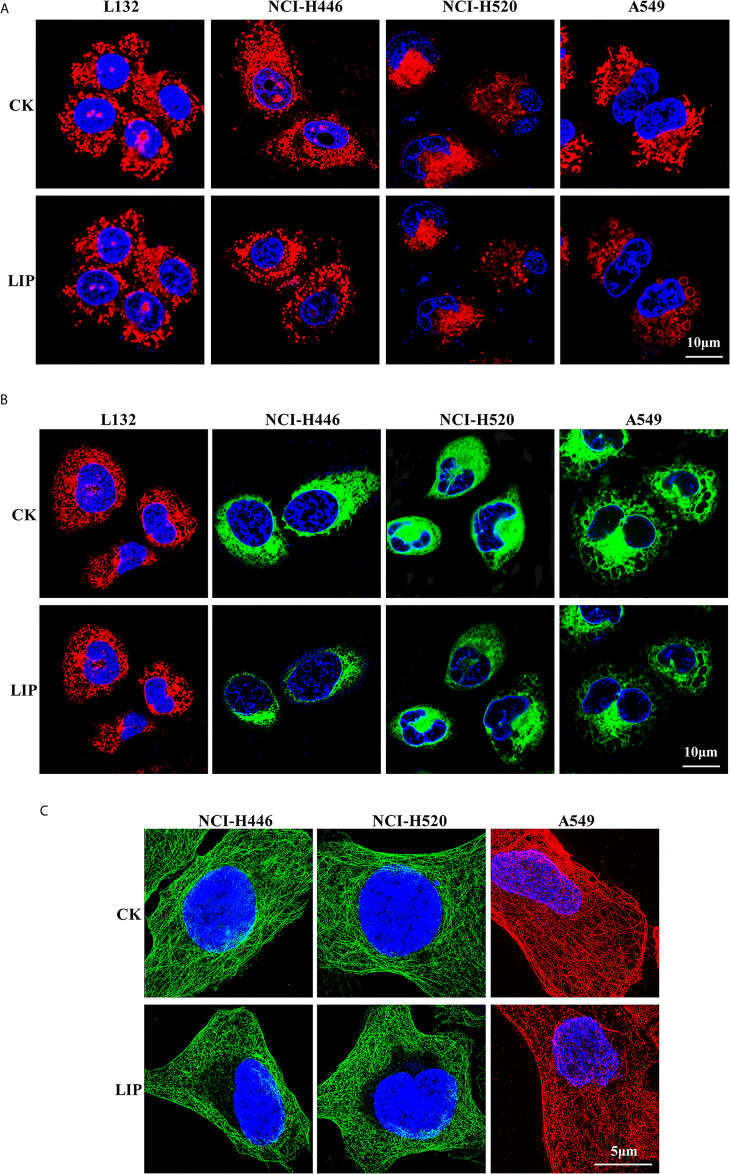
Effect of recombinant LIP on the organelle structure of lung cancer cell lines. **(A)** Effect of LIP on lung cancer cell mitochondria was detected post LIP treatment for 24 h using MitoTracker™ Red CMXRos staining. Hoechst staining was used to detect the nuclei. Scale bar: 10 µm. **(B)** Effect of LIP on the ER of lung cancer cells was detected post LIP treatment for 24 h using ER-Tracker™ Red/Green staining. Hoechst staining was used to detect the nuclei. Scale bar: 10 µm. **(C)** Effect of LIP on the microtubules of lung cancer cells was detected post LIP treatment for 24 h using 3D-SIM imaging with an α-Tubulin Monoclonal Antibody. DAPI staining was used to detect the nuclei. Scale bar: 5 µm.

The results showed that after addition of LIP to lung cancer cells, the mitochondria were wrinkled and compressed into a spherical shape, there was a significant reduction in their number, and their fluorescence disappeared (**Figure 3A**). The ER became swollen and damaged, leading to vacuolation, severe damage to the homeostasis of the cell, and finally to leakage of the cellular contents and apoptosis (**Figure 3B**). The microtubule (observable in the cytoplasm as a diffuse punctate structure) depolymerized, its fluorescence disappeared partially, and was eventually destroyed (**Figure 3C**).

### LIP Induced Apoptosis in Human Lung Cancer Cells

**Figure 4:**
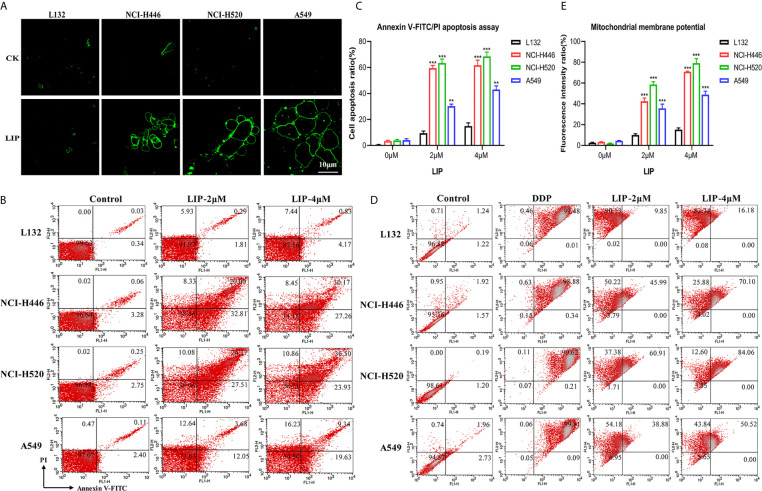
LIP activates the apoptotic pathway in lung cancer cell lines. **(A)** The apoptotic pathway was found to be activated in L132, NCI-H446, NCI-H520, and A549 cells treated with 2 μM LIP for 24 h. Annexin V-FITC staining was used to detect apoptosis. Scale bar: 10 µm. **(B)** The ratio of apoptotic cells was measured in L132, NCI-H446, NCI-H520, and A549 cells following treatment with 2 or 4 μM LIP for 24 h. Apoptosis was detected using Annexin V-FITC/PI staining and flow cytometry. **(C)** Statistical analysis of mortality in the four cell lines. Student’s t-test was used for data analysis; **P < 0.01 and ***P < 0.001, as compared to the control group (n = 3). **(D)** Changes in MMP in lung cancer cells treated with 2 or 4 μM LIP for 24 h. **(E)** Statistical analysis of the MMP in the four cell lines. Student’s t-test was used for data analysis; ***P < 0.001, as compared to the control group (n = 3).

In the present study, Annexin V-FITC, flow cytometry, and other methods were used to detect whether LIP induces apoptosis in human lung cancer cells. Annexin V-FITC staining results showed that LIP generated a greater cytotoxic effect on NCI-H446, NCI-H520, and A549 cells (**Figure 4A**). Under the action of LIP, the cells exhibited an obvious bubbling phenomenon, accompanied by apoptosis, marked by phosphatidylserine (PS) exposure. The green fluorescent point of the circle is the binding site for PS. LIP displayed negligible cytotoxic effects on L132 cells.

According to the results of the flow cytometry experiments (**Figures 4B, C**), following treatment of L132 cells with 2 and 4 μM LIP for 24 h, the cells were in a relatively optimal condition with no obvious apoptosis (0.3 and 0.8%, respectively). After treatment of NCI-H446 cells with 2 and 4 μM LIP for 24 h, the cells displayed obvious apoptosis (20.0 and 30.2%, respectively). In the case of A549 cells, with increasing concentrations of LIP (2 and 4 μM), there was an elevation in the rate of apoptosis (at 3.7 and 9.3%, respectively); however, it was weaker than the corresponding apoptosis rate observed in NCI-H520 and NCI-H446 cells (24.4 and 36.5%, respectively).

As shown in **Figures 4D, E**, L132 cells treated with 2 and 4 μM of LIP for 24 h did not display an obvious decrease in MMP at 9.9 and 16.2%, respectively. In addition, these cells displayed relatively lower green fluorescence intensity. Conversely, in NCI-H446 cells treated with 2 and 4 μM LIP for 24 h, the MMP decreased by 45.9 and 70.1%, respectively. In addition, there was an increase in the green fluorescence intensity, with induction of early apoptosis of the cancer cells. The same treatment caused a decrease in the MMP by 60.9% (2 μM LIP) and 84.1% (4 μM LIP) in NCI-H520 cells, and by 38.9% (2 μM LIP) and 50.5% (4 μM LIP) in A549 cells. These results indicated that LIP destroys the mitochondria and lowers the MMP, thus restricting certain routine cellular activities.

### LIP Induced Lung Cancer Cell Apoptosis *via* the ER Stress Pathway

**Figure 5:**
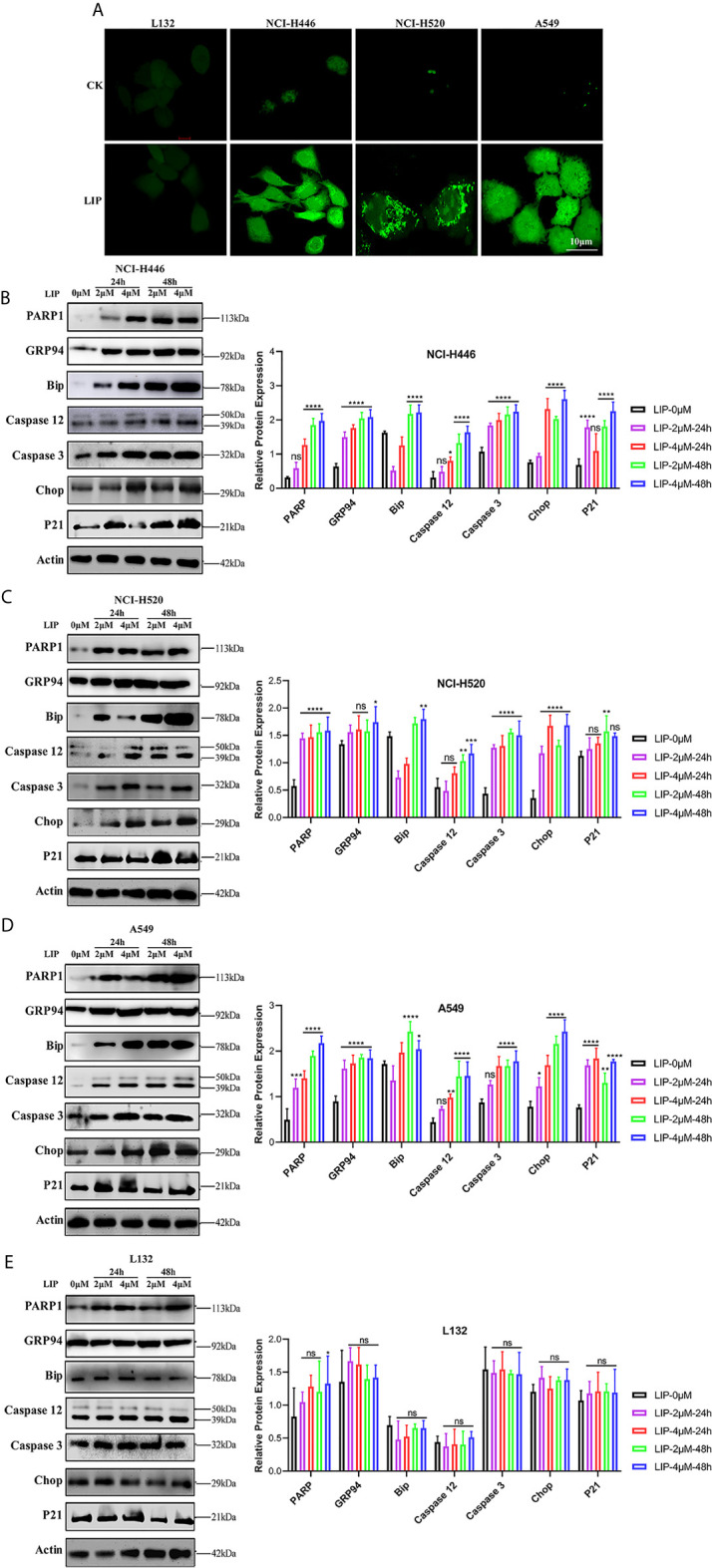
LIP induces apoptosis *via* ER stress. **(A)** Changes in the levels of ROS in lung cancer cells post treatment with 2 μM LIP for 24 h. Scale bar: 100 µm. **(B–E)** Western blot analysis for PARP1, GRP94, Bip, Caspase-12, Caspase-3, CHOP, and p21 expression in NCI-H446, NCI-H520, A549, and L132 cells treated with 2 or 4 μM LIP for 24 h and statistical analysis in the four cell lines. β-actin was used as an internal reference. ImageJ software was used to compare the band densities. Student’s t-test was used for data analysis, ns, not significant; *P < 0.05, **P < 0.01, ***P < 0.001 and ****P < 0.0001, as compared to the control group (n = 3).

To understand whether LIP-mediated destruction of mitochondria is linked to reactive oxygen species (ROS), we examined changes in intracellular ROS levels after LIP treatment. As inferred from the confocal microscope images (**Figure 5A**), the active oxygen levels in NCI-H446, NCI-H520, and A549 lung cancer cells treated with LIP were higher than those in the control group that were not treated with LIP. As compared to lung cancer cells, L132 cells treated with LIP showed no obvious green fluorescence, indicating that there was no significant change in the ROS content in L132 cells.

We further detected the expression of relevant proteins in the ER stress-induced apoptotic pathway using western blot experiments. There was significant treatment time- and dose-dependent upregulation in the expression levels of PARP1, GRP94, Bip, Caspase-12, Caspase-3, CHOP, and p21 in NCI-H446, NCI-H520, and A549 cells treated with 2 or 4 μM LIP for 24 or 48 h (**Figures 5B–D**). Statistical analysis of the expression levels in the four cell lines was shown, wherein ImageJ software was used to compare the band densities in the western blot. After treatment with LIP for 24 and 48 h at different concentrations, there was no significant alteration in the expression levels of PARP1, GRP94, Bip, Caspase-12, Caspase-3, CHOP, and p21 in the L132 cells with increasing LIP concentration (**Figure 5E**). These results implied that ER-induced apoptosis depends on the IRE-1, ATF-6, and PERK pathways, resulting in the upregulation of downstream CHOP expression in lung cancer cells.

**Figure 6:**
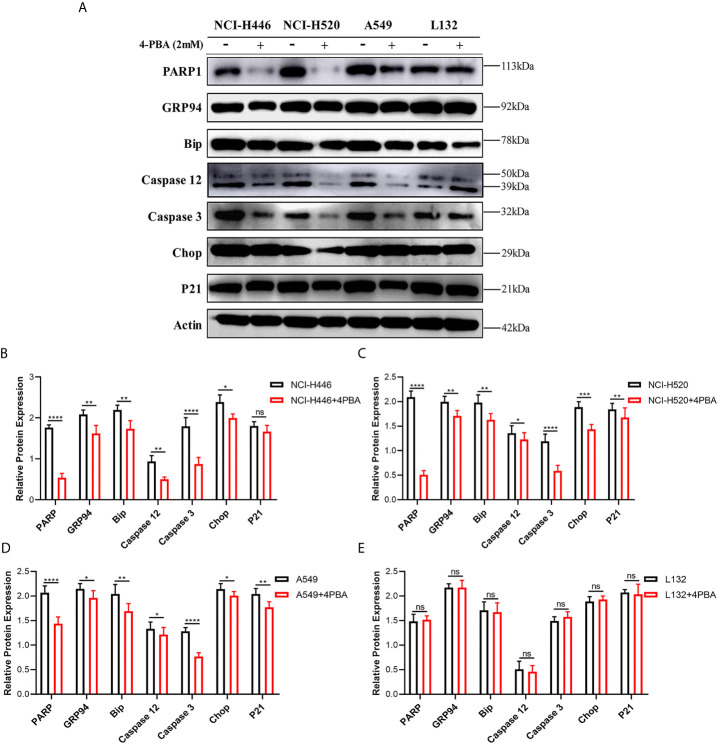
ER stress inhibitor 4-PBA inhibits the ER stress-induced apoptosis mediated by LIP. **(A)** NCI-H446, NCI-H520, A549, and L132 cell lines were treated with 2 mM 4-PBA, an ER stress inhibitor. Thereafter, the expression levels of PARP1, GRP94, Bip, Caspase-12, Caspase-3, CHOP, and p21 in NCI-H446, NCI-H520, A549, and L132 cells treated with 4 μM LIP for 48 h were analyzed using western blot. β-actin was used as an internal reference. **(B–E)** Statistical analysis in the four cell lines. ImageJ software was used to compare the densities of the bands. Student’s t-test was used for data analysis, ns, not significant; *P < 0.05, **P < 0.01, ***P < 0.001 and ****P < 0.0001, as compared to the control group (n = 3).

As shown in **Figure 6A**, upon addition of 2 mM 4-PBA, which is an ER stress inhibitor, to NCI-H446, NCI-H520, A549, and L132 cells treated with 4 μM LIP for 48 h, there was a significant reduction in the expression levels of PARP1, GRP94, Bip, Caspase-12, Caspase-3, CHOP, and p21. The statistical analysis for the western blot experiment in the four cell lines was depicted in **Figures 6B–E**, wherein ImageJ software was used to compare the band densities. p21 is a marker of mitochondrial apoptosis. There was an elevation in the p21 expression level following treatment with LIP, which was attenuated upon treatment with 4-PBA. This indicated that LIP also activates the mitochondrial apoptosis-related pathway, consistent with the conclusion derived from the ROS experiment.

### LIP Acted Synergistically With DDP to Promote Its Cytotoxic Ability in Lung Cancer Cells

**Figure 7:**
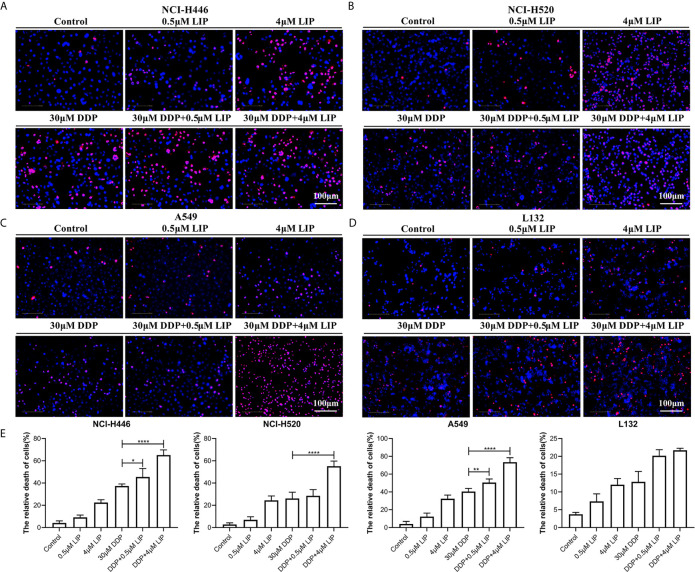
LIP can cooperate with DDP to promote its cytotoxic effect in lung cancer cells. **(A–D)** Mortality of NCI-H446, NCI-H520, A549, and L132 cell lines treated with 0.5 or 4 μM LIP, in combination with/without 30 μM DDP, as analyzed using the Operetta™ High-Content Imaging Analysis System. Scale bar: 100 µm. **(E)** Statistical analysis of mortality in the four cell lines. Student’s t-test was used for data analysis; *P < 0.05, **P < 0.01, ***P < 0.001 and ****P < 0.0001, as compared to the control group (n = 3).

DDP can kill lung cancer cells, but its impact on these cells is limited. It is essential to identify a compound that can act synergistically with DDP to promote its cytotoxic effect in tumor cells. We aimed to verify whether LIP cooperates with DDP to promote the tumoricidal ability of the latter in lung cancer cells. In **Figures 7A–D**, it was shown that NCI-H446, NCI-H520, A549, and L132 cell lines treated with a high dose (4 μM) of LIP, in combination with 30 μM DDP, displayed a higher mortality rate, as compared to cells treated with DDP alone. In addition, a low dose of 0.5 μM LIP was also capable of increasing the mortality rate of DDP in these cells. The statistical analysis of the mortality rate in the four cell lines was depicted in **Figure 7E**.

### LIP Inhibit Lung Cancer Tumor Growth in Nude Mice

**Figure 8:**
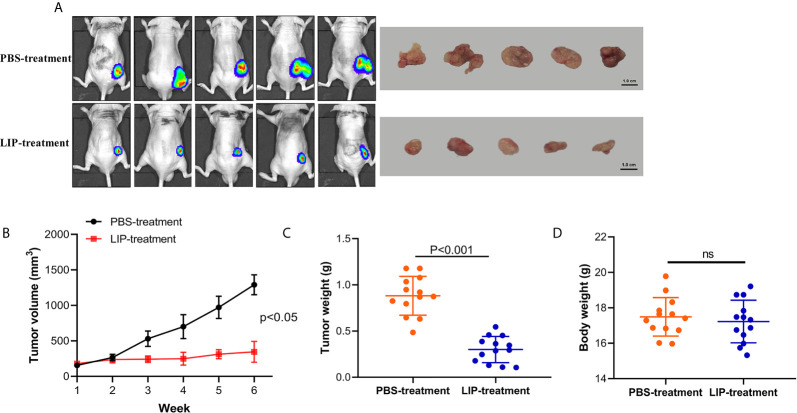
LIP produces potential anti-tumor effects and low toxicity *in vivo*. **(A)** Nude mice with A549-Luc-C8 tumor growths were intratumorally injected with 20 μg/kg LIP or PBS and monitored using the IVIS Lumina Series III *In Vivo* Imaging System, and their tumors were separated from the surrounding muscles at the end of the experiment. **(B)** Tumor sizes in the control and LIP-treated groups (n = 13 per group). **(C)** Weights of tumors in the control and LIP-treated groups (n = 13 per group). **(D)** Body weights in the control and LIP-treated groups (n = 13 per group). ns; not significant.

To understand whether LIP can inhibit lung cancer tumor growth *in vivo*, we established preclinical human A549-Luc-C8 cells tumor models. The mice with tumors received intratumoral injections of LIP into site-specific tumors every 2 days. Mice with tumors received PBS injections as a control. The tumor sizes and changes in body weights of the nude mice were monitored. The results showed that the tumor sizes were significantly lower in the nude mice of the experimental group, as compared to the tumor sizes recorded in the PBS-treated group (**Figures 8A, B**). Furthermore, the average weight was reduced to a quarter of that in the PBS-treated group (**Figure 8C**), and produced no significant effect on the weight of the mice (**Figure 8D**).

### LIP Specifically Recognized and Bound to Human Lung Cancer Tissues

**Figure 9:**
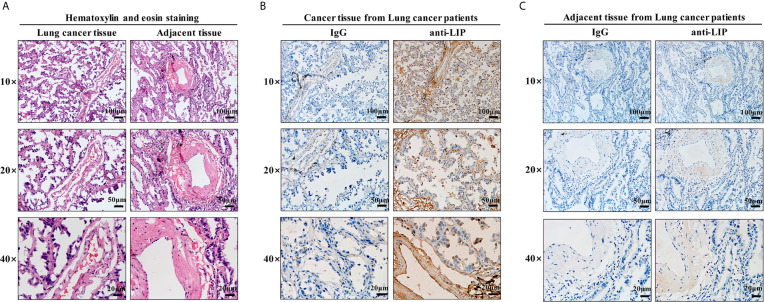
Identification of LIP in tissue sections. **(A)** HE staining of human lung cancer and adjacent tissues. Scale bars: 100 μm, 50 µm, 20 μm. **(B)** Identification capacity of LIP detected using immunohistochemistry of human lung cancer tissue frozen sections. **(C)** Identification capacity of LIP detected using immunohistochemistry of paracancerous tissues of human lung cancer frozen sections. IgG in the figure is an abbreviation for mouse homotypic control.

To identify whether LIP can also be used as a marker to recognize and bind human lung cancer tissues, HE staining and immunohistochemical staining of frozen sections were performed on human lung cancer tissues and adjacent tissues. HE staining results showed that human lung cancer cells exhibited dense cell distribution, imbalanced cytoplasmic nuclear proportion, and enlargement of the nucleus (**Figure 9A**). Immunohistochemical staining results showed that LIP specifically recognizes and binds to lung cancer tissues (**Figure 9B**) but not to adjacent tissues (**Figure 9C**), indicating that the binding ability of LIP to lung cancer cells is specific to the tissues.

## Discussion

The aerolysin domain-containing proteins are widely distributed, from bacteria to vertebrates. Specifically, the aerolysin domain of LIP shows high similarity to the aerolysin domains of other vertebrate proteins, however, LIP contains a jacalin-like lectin domain except for an aerolysin-like pore-forming domain, which can efficiently recognize and destroy target cells by forming transmembrane pores. Several members of this family are bacterial toxins (12). Therefore, it is essential to understand the structural details of the transmembrane pores as well as their mechanism of pore formation for future drug design. The stability of pores and other properties, such as specificity for certain cell-surface molecules, render this family of proteins a useful set of molecular tools for molecular recognition and sensing in cell biology. Currently, a variety of aerolysin nanopore-based identification tools have shown potential for the diagnosis of cancer and other diseases (13, 14). However, the molecular mechanisms by which aerolysin-like pore-forming proteins, especially the non-bacterial aerolysin-like proteins, regulate tumor cell apoptosis have not been thoroughly assessed (15–17). This is important because these proteins are different from simple aerolysin toxins of bacterial origin and may be equipped with more complicated recognition mechanisms (18).

Apoptosis is a representative form of programmed cell death that is induced by various internal and external pathways or ER stress (19, 20). The caspase family plays an indispensable role in the apoptosis signaling pathway (21, 22). In the present study, we found that treatment with LIP activates apoptosis-related pathways in three lung cancer cell lines. Expression levels of the apoptosis-related molecules Bip, CHOP, and Caspase-12 were upregulated in the lung cancer cell lines, whereas no such effect was observed in L132 cells. LIP exerts inhibitory effects on both small-cell lung cancer (NCI-H446) and non-small-cell lung cancer (A549) cell lines. This may be because the recognition target of LIP is not a specific protein, but N-glycolylneuraminic acid (Neu5GC) and sphingomyelin on the tumor surface (18). This also implies that LIP generates a broad-spectrum inhibitory effect on lung cancer cells. Although the results showed that LIP produced an inhibitory effect on all three cell lines (NCI-H520, NCI-H446, and A549), the degree of inhibition was different, with the best inhibitory effect seen in NCI-H520 cells and the worst in A549 cells. This may be related to the Neu5Gc and sphingomyelin content on the surface of the different tumor cells. LIP may exert a relatively weak action on tumors with low levels of Neu5Gc and sphingomyelin (23). MMP analysis demonstrated that LIP can disrupt the mitochondrial membrane structure and lower the MMP. The change in MMP was highest for NCI-H520, while NCI-446 and A549 displayed smaller changes, consistent with the results of the cytotoxic effects of LIP on the three tumor cell lines. The results of the ROS experiment further confirmed tumor cell apoptosis (24, 25).

To elucidate the inhibitory effect of LIP on tumors, we selected nude mice inoculated with A549-Luc-C8 cells for *in vivo* experiments. We observed that LIP treatment inhibited tumor growth in nude mice, as compared to PBS-treated mice; however, it scarcely affected the weights of the mice. However, it is clinically difficult for LIP to directly target tumor tissues (because of its molecular weight of approximately 35 kDa). A possible future research direction could be the fabrication of nanopores for LIP, as demonstrated with other aerolysin proteins (26–28). However, since Neu5Gc is also commonly consumed through a daily diet of milk and red meat, recognition of Neu5Gc by LIP could impact the specificity of LIP recognition (29, 30).

## Conclusion

**Figure 10:**
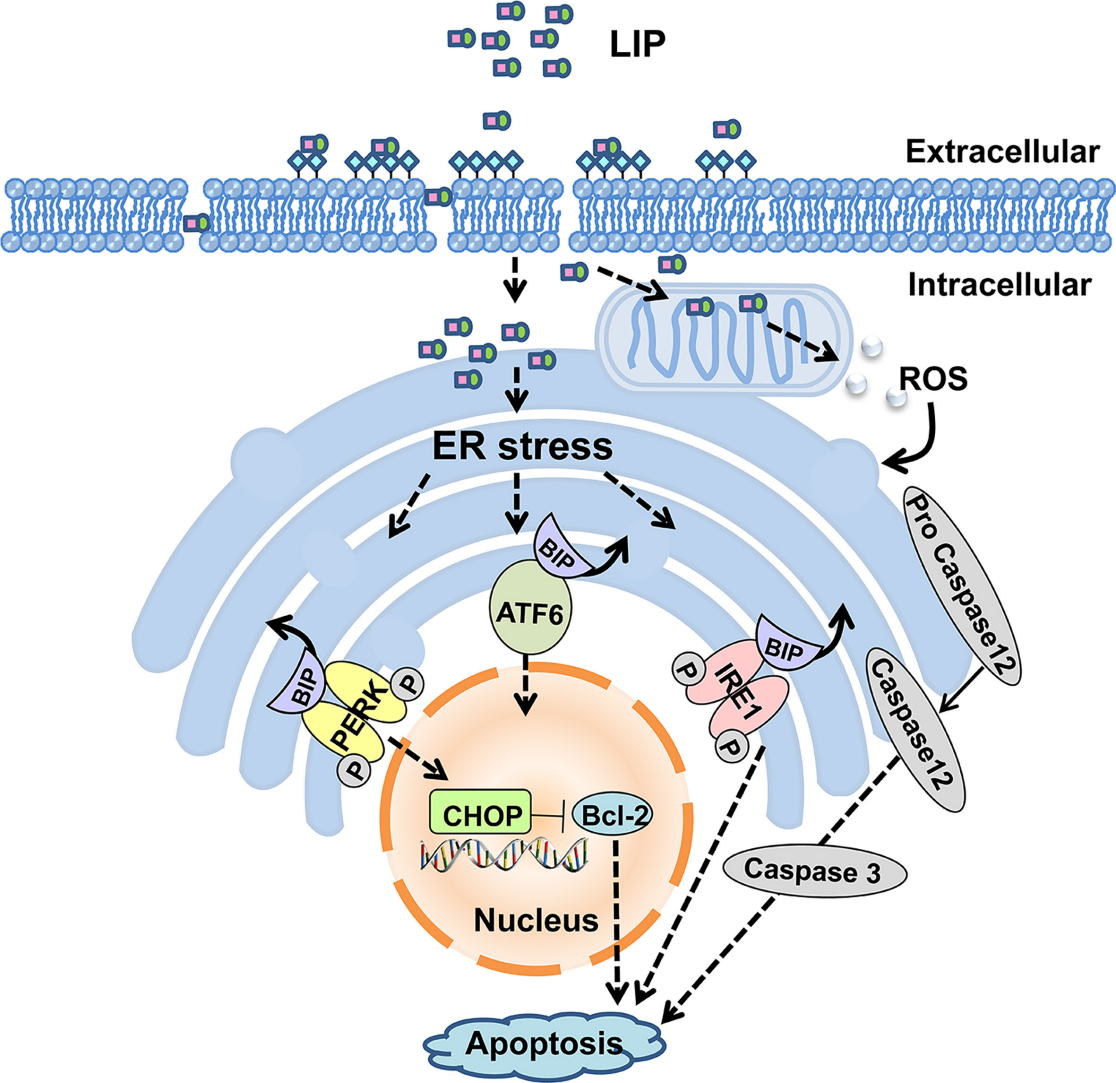
Putative model for LIP-mediated lung cancer cell apoptosis via the ER stress signaling pathway.

In summary, our present study shows that LIP can inhibit the growth of both small-cell lung cancer and non–small-cell lung cancer tumors. Moreover, LIP induces apoptosis of various lung cancer cells through upregulation of the ER stress signaling pathway. The model for LIP-mediated regulation of lung cancer cell apoptosis is briefly illustrated in **Figure 10**. LIP can be employed as a target for the direct treatment of lung cancer or as a platform for the delivery of anti-cancer drugs. Thus, it can serve as a promising strategy for clinical diagnosis and treatment of lung cancer patients.

## Data Availability Statement

The raw data supporting the conclusions of this article will be made available by the authors, without undue reservation.

## Ethics Statement

The animal study was reviewed and approved by NIH Publications No. 8023, revised 1978. Written informed consent was obtained from the individual(s) for the publication of any potentially identifiable images or data included in this article.

## Author Contributions

YP and QL led study design and prepared the manuscript. XS and XX carried out the experiments. JL and XC assisted in tissue sample collection and data analysis. All authors contributed to the article and approved the submitted version.

## Funding

This work was funded by the Chinese National Natural Science Foundation Grants (No.31772884, No.32070518). The project of Department of Ocean and Fisheries of Liaoning Province (No.201805), Program of Science and Technology of Liaoning Province (No.2019-MS-218), Science and Technology Innovation Fund Research Project (No. 2018J12SN079) and Liaoning Climbing Scholar, the Distinguished Professor of Liaoning (NO.XLYC2002093).

## Conflict of Interest

The authors declare that the research was conducted in the absence of any commercial or financial relationships that could be construed as a potential conflict of interest.
